# Multi-Objective Parameter Optimization Design of Heat Pipe Heat Sink for Bidirectional Power Converter Based on MOEDO Algorithm

**DOI:** 10.3390/mi17050514

**Published:** 2026-04-23

**Authors:** Zechen Su, Xiwei Zhou, Yangfan Li, Qisheng Wu, Hongwei Zhang, Binyu Wang, Weiyu Liu

**Affiliations:** School of Electronic and Control Engineering, Chang’an University, Xi’an 710064, China

**Keywords:** MOEDO (Multi-Objective Exponential Distribution Optimizer), bidirectional power converter, heat sink, multi-objective optimization, response surface surrogate model

## Abstract

Bidirectional power converters generate significant heat losses during high-frequency operation, posing a severe challenge to the performance of heat dissipation systems. Traditional thermal design methods often struggle to balance multiple objectives, such as cooling efficiency, cost, weight, and size, thereby limiting the reliability and safety of the system. To address these challenges, this paper proposes a novel Multi-Objective Exponential Distribution Optimizer algorithm based on the Exponential Distribution Optimizer. Subsequently, key design variables of the heat dissipation system are selected. Next, the Optimal Latin Hypercube Sampling method is employed to generate sample points, and a second-order response surface surrogate model for the heat pipe radiator’s volume and temperature is developed. Lastly, by integrating elite non-dominated sorting, crowding distance mechanisms, and an information feedback mechanism, the multi-objective challenge is decomposed into subproblems, thereby enhancing optimization efficiency. Through comparative simulation experiments on benchmark functions, the Wilcoxon signed-rank test results for the MOEDO algorithm on the majority of the three metrics are denoted as ‘+’, indicating statistically significant advantages over the compared algorithms, thereby demonstrating its superior performance in addressing multi-objective optimization problems. The study further conducts simulation verification of the heat pipe heat dissipation system before and after optimization using ANSYS Icepak. The simulation results demonstrate that, compared with the conventional design, the maximum Insulated Gate Bipolar Transistor (IGBT) temperature is reduced by 17.12% and the heat sink volume is reduced by 14.61%.

## 1. Introduction

The bidirectional power converter is a technologically advanced device capable of converting direct current (DC) power to alternating current (AC) power or vice versa [1]. However, in addition to its functionality, the heat generated during operation poses a significant challenge. As an essential core component in modern power electronics systems, the bidirectional power converter produces substantial heat losses due to the frequent switching of its internal power devices during high-frequency operation. This heat accumulation not only severely impacts the converter’s operational efficiency but also affects its long-term stability and safety. The design of the cooling system must comprehensively consider multiple factors to ensure effective heat dissipation while minimizing weight and space occupancy. In such problems, different objectives often conflict and require trade-offs, wherein optimizing one objective may compromise the performance of another [2].

Traditional multi-objective optimization algorithms, such as NSGA-II, SPEA2, MOPSO, and MOEA/D, have been widely applied in thermal management problems. For instance, NSGA-II demonstrates excellent solution diversity by providing good Pareto front coverage in applications like optimizing segmented thermoelectric generators for maximum efficiency and minimum volume or the three-objective optimization of solar ejector cooling systems to achieve optimal thermal efficiency and operating conditions. However, NSGA-II exhibits medium convergence speed, often requiring multiple iterations for complex three-dimensional models, and is susceptible to local optima in non-convex fronts, particularly in large-scale problems. In comparison, SPEA2 offers good diversity but suffers from higher computational complexity, making it less suitable for real-time thermal applications. MOPSO provides faster convergence but is prone to particle stagnation and high susceptibility to local optima, leading to poor coverage in extreme Pareto regions, as seen in heat exchanger optimizations. MOEA/D achieves fast convergence with low susceptibility to local optima through decomposition, excelling in high-dimensional problems, but its performance is sensitive to weight distribution, resulting in uneven solution sets in cases with uneven heat distributions [3]. As evidenced in specific studies, NSGA-II reduced a combined function of temperature difference and pumping power by 25.7% in composite cooling channels [4] and sought Pareto solutions for Nusselt number and friction coefficient under a Reynolds number of 49,013 in helical microfins [5]. However, these applications highlight its inefficiencies in convergence speed and its susceptibility to local optima in non-convex problems limit its efficiency for large-scale thermal designs.

The Exponential Distribution Optimizer (EDO) leverages the properties of exponential distribution to conduct global searches, demonstrating rapid convergence. However, the standard EDO often fails to maintain balance among various objectives in multi-objective problems and is prone to disruptions from local optima. This study addresses these limitations by proposing the Multi-Objective Exponential Distribution Optimizer (MOEDO), which integrates elite non-dominated sorting, crowding distance, and information feedback mechanisms into the EDO framework. This extension is necessary to overcome the slow convergence and local optima issues of traditional algorithms like NSGA-II in thermal design, thereby providing superior performance in optimizing heat pipe heat sinks for bidirectional power converters.

The primary contributions of this work, advancing beyond traditional multi-objective algorithms that often suffer from slow convergence and local optima in thermal design applications, are as follows: (1) the selection of six key geometric variables for the heat pipe heat sink, with engineering constraints derived from thermal resistance analysis and practical standards [6]; (2) the construction of a second-order response surface surrogate model using Optimal Latin Hypercube Sampling (OLHS) with 28 points for the efficient prediction of radiator volume and maximum IGBT temperature; (3) the proposal of the MOEDO algorithm, extending the single-objective EDO by incorporating elite non-dominated sorting, crowding distance, and an information feedback mechanism to enhance convergence, diversity, and avoidance of local optima; (4) validation through benchmark tests and ANSYS Icepak 2025 R2 simulations, demonstrating superior performance and simultaneous improvements in thermal efficiency and compactness. The remainder of this paper is organized as follows: Section 2 establishes the mathematical model and design specifications; Section 3 details the MOEDO algorithm; Section 4 presents algorithm performance comparisons; Section 5 reports experimental results and analysis; and Section 6 concludes the study.

## 2. Mathematical Model Construction

### 2.1. Design Specifications

The design of a heat pipe heat sink for a bidirectional power converter requires the identification of an optimal compromise among multiple conflicting objectives to meet demanding and highly variable operating conditions. This study defines two primary optimization objectives: maximum IGBT temperature and radiator volume. The IGBT is a core power device in the bidirectional power converter. It incurs significant heat losses from conduction and switching. These losses cause rapid heating and a rapid increase in module temperature. Prolonged exposure to high temperatures subjects the IGBT module to severe thermal stress, leading to performance degradation, faults, and damage. Radiator volume is the sum of the radiator base and fin volumes excluding portions where heat pipes are embedded. Reducing volume reduces material costs and manufacturing complexity, thereby enhancing the economic viability of the system.

A thermal resistance network analysis indicates that numerous geometric parameters potentially influence heat dissipation performance, volume, and weight. To ensure a well-focused optimization problem and practical engineering feasibility, fin height and the number of heat pipes are deliberately excluded from the design variables and treated as fixed parameters: fin height is constrained to the conventional range of 40–63 mm, which appropriately balances the diminishing convective heat-transfer gains under natural convection; beyond this range, taller fins yield only marginal cooling improvement while significantly increasing volume. Likewise, adding more heat pipes considerably elevates manufacturing complexity and cost, yet provides limited additional thermal benefit and complicates assembly. Consequently, both fin height and the number of heat pipes are not considered as optimization variables in this study.

Based on the above considerations, six parameters that exert the most significant and simultaneous effects on thermal resistance, cooling capacity, volume, and weight are selected as the design variables: baseplate length, baseplate width, fin thickness, fin spacing, total heat pipe perimeter, and heat pipe radius. The maximum IGBT junction temperature and the heat sink volume (defined as the combined volume of the baseplate and fins, excluding the portions occupied by embedded heat pipes) are adopted as the two conflicting optimization objectives.

To ensure that all candidate designs are practically viable, engineering constraints are imposed on these variables. The bounds are comprehensively derived from converter enclosure space limitations, typical IGBT module layouts, aluminum extrusion process capabilities, natural-convection airflow characteristics, and heat pipe bending and embedding specifications.

(1) $$\left\{\begin{array}{l} 160\;\mathrm{mm}\leq a_{1}\leq 200\mathrm{mm}\\ 130\;\mathrm{mm}\leq a_{2}\leq 160\mathrm{mm}\\ 1\;\mathrm{mm}\leq b\leq 5\mathrm{mm}\\ 1\;\mathrm{mm}\leq d\leq 5\mathrm{mm}\\ 1937\;\mathrm{mm}\leq c\leq 2094\mathrm{mm}\\ 3\;\mathrm{mm}\leq r\leq 4.5\mathrm{mm} \end{array}\right.$$

In the above constraints, *a*_1_ is the base length, *a*_2_ is the base width, *b* is the fin thickness, *d* is the fin spacing, *c* is the heat pipe circumference, and *r* is the heat-pipe radius. The bounds of the design variables are collectively determined by practical engineering constraints to ensure structural integrity, manufacturing feasibility, thermal performance, and compatibility with the limited installation space of the converter enclosure. Specifically, the base length *a*_1_ and width *a*_2_ are restricted to 160–200 mm and 130–160 mm, respectively, to provide adequate heat-spreading area for multiple IGBT modules while remaining compatible with standard rack-mount dimensions. The fin thickness b and fin spacing d are both limited to the range of 1–5 mm, as values below 1 mm fail to satisfy the minimum structural strength required for aluminum extrusion and assembly, whereas values exceeding 5 mm either excessively reduce the effective heat-transfer surface area or unnecessarily increase the radiator volume without a commensurate improvement in airflow. The total heat pipe circumference c is confined to 1937–2094 mm to supply the embedded length necessary for achieving reasonably uniform temperature distribution across the base, and the heat pipe radius r is set between 3 mm and 4.5 mm to comply with the industry-standard minimum bending radius guideline (greater than three times the diameter) that prevents damage to the internal sintered wick while avoiding excessive material consumption. Therefore, the constraint range for base length a_1_ is set from 160 mm to 200 mm; the constraint range for base width a_2_ is set from 130 mm to 160 mm; the constraint range for fin thickness b is set from 1 mm to 5 mm; the constraint range for fin spacing d is set from 1 mm to 5 mm; the constraint range for heat pipe circumference c is set from 1937 mm to 2094 mm; and the constraint range for heat pipe radius r is set from 3 mm to 4.5 mm.

### 2.2. Deterministic Optimization Design

**Figure 1:**
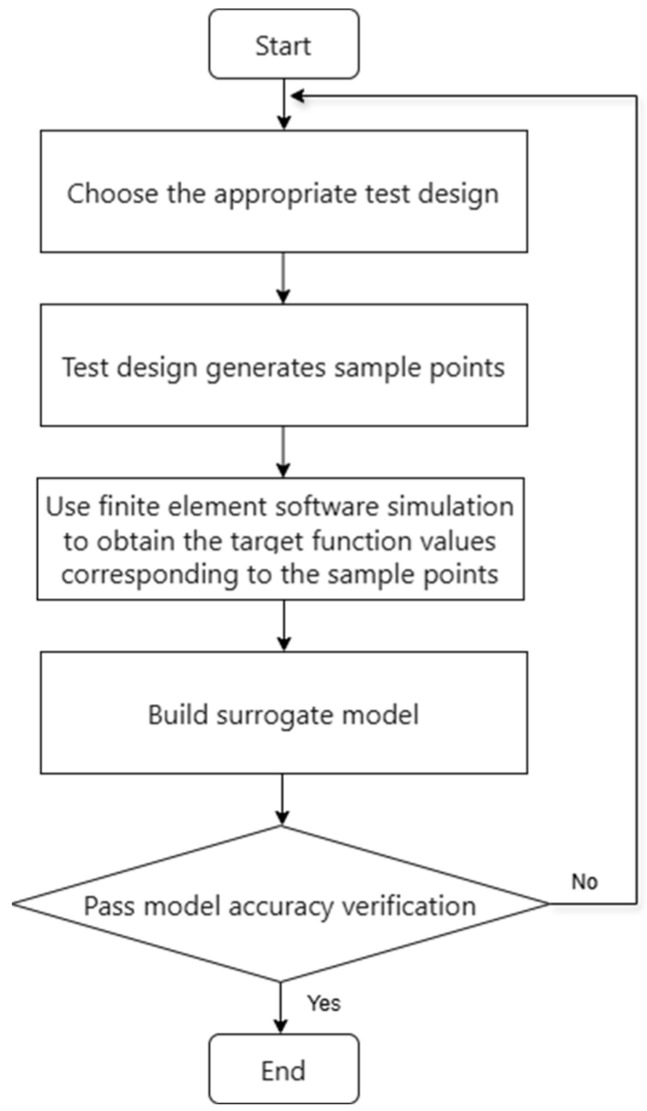
Multi-objective optimization flowchart.

When establishing the initial model, to meet design requirements, certain variables need to be optimized. Figure 1 illustrates the overall process of deterministic optimization design, which is typically divided into four main parts.

Firstly, according to the performance requirements of the heat dissipation system, the design variables and optimization goals of the heat dissipation system are determined.

Next, the optimal Latin hypercube sampling (OLHS) method is used to generate sample points.

Subsequently, for each sample point, the finite element simulation software was used to calculate the objective function values such as radiator volume and temperature rise.

Thirdly, sample points were fitted with the corresponding target response data to establish a mathematical mapping relationship between the volume of the heat dissipation system and the temperature rise.

Finally, the model is evaluated to ensure that its prediction accuracy meets the optimization requirements; otherwise, the experimental design needs to be adjusted or the sample points need to be added for improvement.

### 2.3. Construction of the Response Plane Proxy Model

When analyzing the heat sink design, there is a significant contradiction between reducing the size and improving the heat dissipation efficiency. By considering design variables such as substrate length, substrate width, fin thickness, and fin spacing, a balance is found between ensuring sufficient heat dissipation area and optimizing air flow to achieve efficient heat dissipation while controlling the overall size of the heat sink.

**Figure 2:**
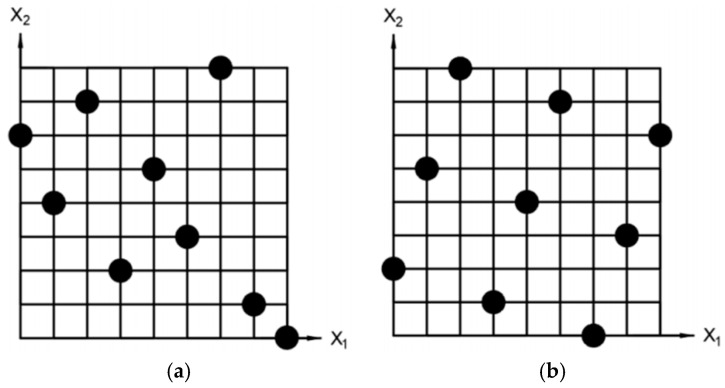
Schematic diagrams of LHS and OLHS spaces. (**a**) LHS; (**b**) OLHS.

A response surface surrogate model is constructed to predict heat sink volume and maximum IGBT junction temperature. To generate high-fidelity training data for the subsequent multi-objective optimization, Optimized Latin Hypercube Sampling (OLHS) is adopted. Schematic diagrams of the LHS and OLHS spaces are presented in Figure 2.

Although standard LHS guarantees stratification in each marginal dimension, random permutation may still introduce undesirable inter-variable correlations. OLHS overcomes this limitation by initializing from a standard LHS and subsequently applying a global optimization algorithm (e.g., genetic algorithm or enhanced stochastic evolutionary algorithm) to refine sampling point locations while preserving the Latin hypercube property. Compared with standard LHS, OLHS typically reduces the maximum pairwise correlation coefficient from 0.12–0.38 to below 0.05 and improves centered L2-discrepancy and the φ_50_ criterion by 30–70% [7,8]. These enhancements yield a significantly higher surrogate model accuracy for a given sample size, thereby reducing the overall computational burden of the optimization process.

The relationship between the target variable and the design variable of the second-order polynomial response surface model can be expressed as follows:

(2) $$f(x)=a_{0}+\sum_{i=1}^{N}b_{i}x_{i}+\sum_{ij(i<j)}c_{ij}x_{i}x_{j}+\sum_{i=1}^{N}d_{i}x_{i}^{2}$$

*f*(*x*) represents the target variable, *N* represents the number of design parameters to be optimized, *x_i_* and *x_j_* represent the *i*-th and *j*-th components of the input variable, respectively, *a*_0_ represents the intercept term, *b_i_* represents the linear coefficient, *c_ij_* represents the interaction coefficient, and *d_i_* represents the quadratic coefficient.

The minimum number of sample points required for the second-order response surface surrogate model is determined by the following formula:

(3) $$N_{\min}=\frac{\left(n+1)(n+2\right)}{2}$$

*N*_min_ represents the number of design variables, ensuring that the model has enough degrees of freedom to estimate all coefficients. This equation ensures sufficient degrees of freedom to uniquely estimate all linear, interaction, and quadratic coefficients in Equation (2).

There are six design variables in this paper: substrate length, substrate width, fin thickness, fin spacing, heat pipe radius, and heat pipe perimeter. Therefore, in the response surface method, in order to construct a second-order model, the number of sample points obtained in Equation (3) is 28. The coefficient of determination *R*^2^ was estimated using the standard formula for model adequacy assessment:

(4) $$R^{2}=1-\frac{\sum_{i=1}^{n}{\left(y_{i}-{\hat{y}}_{i}\right)}^{2}}{\sum_{i=1}^{n}{\left(y_{i}-\bar{y}\right)}^{2}}$$

where $\sum_{i=1}^{n}{\left(y_{i}-{\hat{y}}_{i}\right)}^{2}$ is the residual sum of squares and $\sum_{i=1}^{n}{\left(y_{i}-\bar{y}\right)}^{2}$ is the total sum of squares. The second-order polynomial surrogate model was fitted to the OLHS sample points via ordinary least-squares regression.

To determine the appropriate scale for the surrogate model, the fitting precision was rigorously assessed using quantitative metrics. For the second-order response surface model, based on 28 sample points, the coefficient of determination *R*^2^ for the maximum temperature reached 0.9921 with a Root Mean Square Error (RMSE) of 0.428 °C, while for the radiator volume, the *R*^2^ was 0.9992, with an RMSE of 1.05 × 10^−6^ m^3^.

To evaluate the sensitivity of model accuracy to sample size, additional models with 50 and 100 sample points were further tested. The results indicate that increasing the sample size to 100 only improved the *R*^2^ of T_max_ from 0.9921 to 0.9942, with negligible improvements in RMSE. This quantitatively demonstrates that the initial 28-point model possesses high fitting fidelity, effectively capturing the complex non-linear relationships between design variables and objective functions while achieving an optimal balance between computational efficiency and predictive accuracy.

The sample points are shown in Table 1.

## 3. MOEDO Algorithm

In the multi-objective optimization problem of heat dissipation system, although the traditional multi-objective optimization algorithm (such as NSGA-II) can find the Pareto solution set to a certain extent, it often has problems such as local optimum, slow convergence speed and insufficient diversity of solution set. In order to solve these shortcomings, based on the EDO algorithm, this paper proposes a Multi-objective Exponential Distribution Optimizer (MOEDO) algorithm integrating Non-Dominance Sorting (NDS), Crowding Distance (CD) and Information Feedback Mechanism (IFM) to decompose the multi-objective challenge into single-objective subtasks and improve the efficiency of the algorithm. The dynamics of utilizing the IFM approach to ensure a balance between exploration and development promote improved convergence and the ability to bypass local minima.

### 3.1. MOEDO Algorithm Design

The EDO algorithm leverages the properties of exponential distribution to conduct global searches in the solution space, demonstrating a rapid convergence rate. However, the standard EDO often fails to maintain a balance among various objectives in multi-objective problems and is prone to disruptions from local optima. To tackle these challenges, the MOEDO algorithm incorporates the following three mechanisms based on EDO: Non-Dominated Sorting (NDS), Crowding Distance (CD), and Information Feedback Mechanism (IFM).

(1) Non-dominated Sorting (NDS)

**Figure 3:**
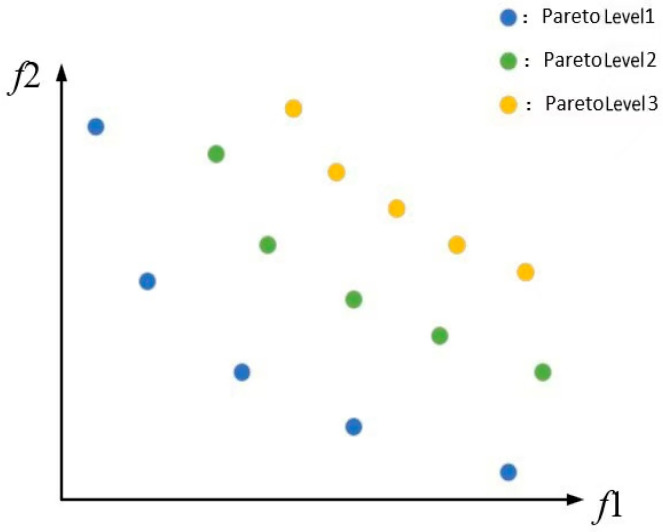
Schematic diagram of Pareto grades.

The objective of non-dominated sorting is to categorize a set of solutions into multiple levels, where the solutions within each level do not dominate one another, and solutions with higher priority are placed in earlier levels. The fast non-dominated sorting in MOEDO is an efficient algorithm for partitioning solutions into several levels. Based on the dominance relationships, the solution set can be divided into various layers of Pareto fronts. The first layer consists of solutions that are not dominated by any other solutions, the second layer contains solutions that are only dominated by those in the first layer, and so forth. This creates a series of Pareto front layers. A schematic representation of the Pareto ranking is illustrated in Figure 3.

(2) Low Congestion Screening Strategy

The purpose of the low-crowding degree selection strategy is to maintain better diversity within the population. In order to differentiate the sparsity of solutions within the same Pareto frontier level, it is necessary to introduce crowding distance. The crowding distance refers to the average distance between a solution and its neighboring solutions within that Pareto frontier level. The method of calculating crowding distance typically considers the differences in the values of each solution across various objective functions; solutions with greater distances will have larger crowding distances.

The final crowding distance *CD_i_* for individual *i* is the sum of the contribution distances CD*_i,m_* across all M objective functions:

(5) $$CD_{i}=\sum_{m=1}^{M}CD_{i,m}$$

where the contribution distance *CD_i,m_* of individual *i* on objective function *m* is calculated using the formula:

(6) $$CD_{im}=\frac{f_{m}(x_{i+1})-f_{m}(x_{i-1})}{f_{m}(x_{\max})-f_{m}(x_{\min})},i=2,\ldots ,(l-1)$$

*CD_i,m_* represents the contribution to the Crowding Distance of the *i*-th individual with respect to the *m*-th objective function. *f_m_* denotes the *m*-th objective function. *X*_max_ refers to the maximum *f_m_* value observed among all individuals in the current front and *X*_min_ refers to the minimum *f_m_* value.

**Figure 4:**
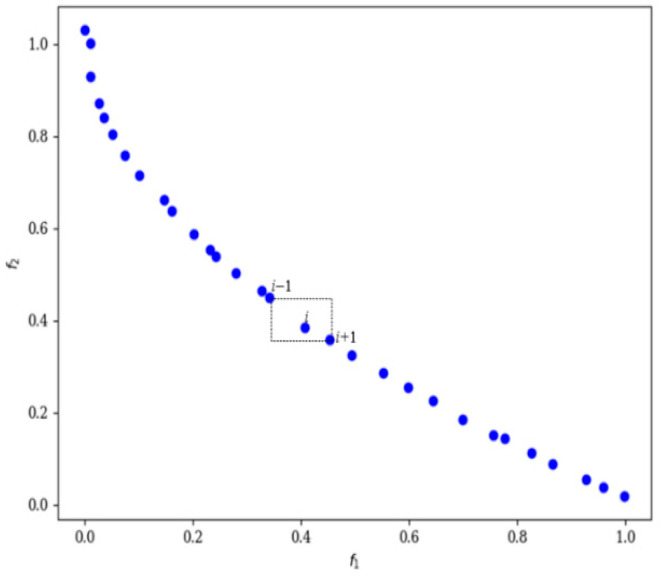
Pareto frontier curves of the same grade.

To illustrate this more clearly, we can refer to a specific level of the Pareto frontier with two objective functions, as shown in Figure 4. The two individuals at the left and right boundaries are assigned infinite crowding distances *d* = ∞,while the crowding distance of individual i is defined as the perimeter of a rectangle formed by using individual *i* − 1 as the upper left vertex and individual *i* + 1 as the lower right vertex, denoted as d_i_. The higher the value of *d_i_*, the greater the crowding distance, leading to a lower degree of crowding.

(3) Information feedback mechanism (IFM)

The Information Feedback Mechanism (IFM) serves as a core fusion operator within the MOEDO algorithm, designed to dynamically balance the algorithm’s Exploration and Exploitation capabilities in a multi-objective environment. IFM generates new offspring solutions by fusing information from the candidate solution U_i_ with the current parent solution $x_{i}^{t}$ through weighting coefficients ∂_1_ and ∂_2_. The key to this mechanism lies in determining these coefficients: this algorithm employs the Fitness-Driven Dynamic Weighting Method. These dynamic weights are determined based on a Composite Fitness Score S (X) for both the candidate and parent solutions, a score which effectively integrates the non-dominated rank and the crowding distance mechanism. This choice is highly justifiable: firstly, it adapts well to multi-objective optimization scenarios by directly assigning weights based on the intrinsic quality of the solution, which is superior to methods like AHP, which assign weights based on objective importance (objective space). Secondly, its computational complexity is controllable as the weight calculation relies solely on existing rank and CD values, thereby avoiding the additional computational overhead required by complex external tools and ensuring that IFM enhances the algorithm’s performance while efficiently achieving an adaptive balance between exploration and exploitation.

### 3.2. MOEDO Algorithm Process

The Exponential Distribution Optimizer (EDO) is a population-based metaheuristic algorithm primarily designed for single-objective continuous optimization problems. It employs an iterative update mechanism that uses a random threshold to effectively balance exploitation and exploration throughout the search process.

The flow chart of EDO algorithm is shown in Algorithm 1.

Based on the EDO algorithm, the MOEDO algorithm begins with a random population of size N. Let the current generation be denoted as *t*, where $x_{i}^{t}$ represents the *i*-th individual of the *t*-th generation, and $x_{i}^{t+1}$ represents the *i*-th individual of the (*t* + 1)-th generation. The individual $U_{i}^{t+1}$ is generated for the (*t* + 1)-th generation from the EDO algorithm and the parent population. The fitness value of $U_{i}^{t+1}$ is denoted as $f_{i}^{t+1}$, and $U^{t+1}$ represents the collection of $U_{i}^{t+1}$. Subsequently, based on the individuals $U_{i}^{t+1}$ generated through the EDO algorithm and the information feedback mechanism, $x_{i}^{t+1}$ can be calculated as follows:

(7) $$\begin{array}{c} x_{i}^{t+1}=\partial_{1}x_{k}^{t}+\partial_{2}x_{k}^{t};\\ \;\partial_{1}=\frac{f_{k}^{t}}{f_{i}^{t+1}+f_{k}^{t}},\;\partial_{2}=\frac{f_{i}^{t+1}}{f_{i}^{t+1}+f_{k}^{t}},\;\partial_{1}+\partial_{2}=1 \end{array}$$

**Algorithm 1.** The pseudocode of the proposed EDO***Input:***  *population size (N), maximum time (Max_Time), dimension (d), lower bound (lb) and upper bound (ub).* ***Output:*** *The optimal solutions obeying the exponential solution (Xwinners_best_)* *1. Initialize a population of N solutions representing the multiple Exponential Distribution models Xwinners_i_ (i = 1, 2, …, N)* *2. Define Fitness vector to store the fitness of all solutions obeying the Exponential Distribution* *3. Find the best fitness (bestfitness) and Xwinners_best_* *4. time = 1* *5. Construct the memoryless matrix such that **memoryless = Xwinners*** *6. **while** (time < Max_Time)* *7.     Define V matrix of size s* *8.     Rank the solutions in Xwinners population in ascending order of their fitness values* *9.     Calculate Xguide^time^ = (*$Xwinners_{best1}^{TIME}$ + $Xwinners_{best2}^{TIME}$ + $Xwinners_{best3}^{TIME}$*)/3* *10.   Define the EDO adaptive parameters a, b, c, d and f* *11.   **for** i = 1:N* *12.      **if** (α < 0.5)//----------------**Apply exploitation phase**----------------* *13.        **if** (Xwinners_i_^time^ == memoryless_i_^time^)* *14.         Update V_i_^time+1^ = a.(Xwinners_i_^time^ − σ^2^) + b.Xguide^time^* *15.      **else*** *16.         Generate φ ∈* [0, 1] *17.         Update V_i_^time+1^ = b.(Xwinners_i_^time^ − σ^2^) + log(φ).Xwinners_i_^time^* *18.        **end*** *19.     **else**//----------------**Apply exploration phase**----------------* *20.       Update V_i_^time+1^ = Xwinners_i_^time^ − M^time^ + (c.Z*_1_ *+ (1 − c).Z*_2_*)* *21.     **end*** *22.     Check bounds of V_i_^time+1^* *23.     **end for*** *24.     Copy V to memoryless matrix* *25.     Define newFitness vector to store the fitness of all solutions of memoryless matrix* *26.     **for** i = 1:N* *27.    **if** (newFitness_i == Fitness_i)* *28.        Update Xwinners_i_ = V_i_ and Fitness_i_ = newFitness_i_* *29.        **if** (Fitness_i < bestfitness)* *30.        Update Xwinners_best_ = Xwinners_i_ and bestfitness = Fitness_i_* *31.     **end if*** *32.   **end if*** *33.   **end for*** *34.   time++* *35. **end while*** *36. **return** Xwinners_best solution*

In Equation (5), $x_{k}^{t}$ represents the *k*-th individual selected from generation *t*, the fitness value of $x_{k}^{t}$ is $f_{k}^{t}$, and ∂_1_ and ∂_2_ are the weight coefficients. A progeny population Q_t_ was generated, where Q_t_ is a collection of $x_{k}^{t+1}$. The combined populations R_t_ = P_t_ ∪ Q_t_ were sorted according to different non-dominance levels (F_1_, F_2_, …, F_l_, …, F_w_). Starting with F_1_, all instances in levels 1 to l were added to the S_t_, and the remaining Rt instances were discarded.

If |S_t_|= N, no further actions are required, and we can directly proceed to the next generation, that is, P_t+1_ = S_t_. Otherwise, the solutions in S_t_/F_1_ are included in P_t+1_, and the remaining solutions are selected from F_1_ based on the crowding distance mechanism. The method of solution selection is determined by the crowding distance of the solutions in F_1_, with a larger crowding distance resulting in a higher probability of selection. We then check whether the termination condition is met. If the termination condition is not satisfied, then t = t + 1, and the process is repeated. If the termination condition is satisfied, P_t+1_ is generated and then applied to the new population Q_t+1_, generated by the EDO algorithm.

The primary computational overhead in the proposed MOEDO algorithm stems from the fast non-dominated sorting procedure, which has a time complexity of O(MN^2^) per generation, where M is the number of objectives and N is the population size. The crowding distance calculation contributes O(MN log N), while the EDO update rules and the information feedback mechanism (IFM) introduce only linear overhead O(ND), where D is the dimension of the decision space.

## 4. Algorithm Simulation Experiments

In this paper, ZDT1, ZDT2, ZDT3, ZDT6 and DTLZ2 are selected as the test functions, ZDT1 is the dual-objective convex function, ZDT2 is the dual-objective concave function, ZDT3 is the dual-objective discontinuous convex function, ZDT6 is the dual-objective concave function, and DTLZ2 is the three-objective function (high-dimensional function). The specific function expressions are shown in Table 2.

In order to ensure the robustness of the results, during the algorithm validation phase, the population size was set to 100 and the number of iterations to 100. The experimental environment of the testing platform is as follows:

(1) CPU: Core(TM) i7 1.80 GHz;
(2) RAM: 8 GB;
(3) Operating System: Windows 10;
(4) Programming Language: Python 3.14.0.

**Figure 5:**
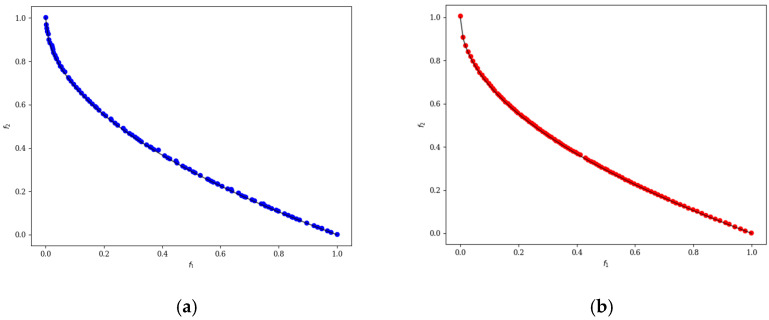
ZDT1 Pareto curve: (**a**) NSGA-II; (**b**) MOEDO.

**Figure 6:**
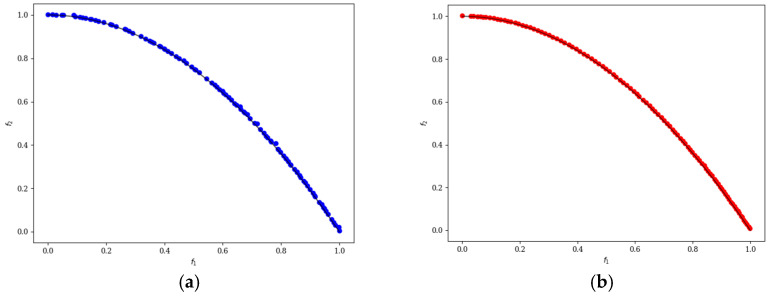
ZDT2 Pareto curve: (**a**) NSGA-II; (**b**) MOEDO.

**Figure 7:**
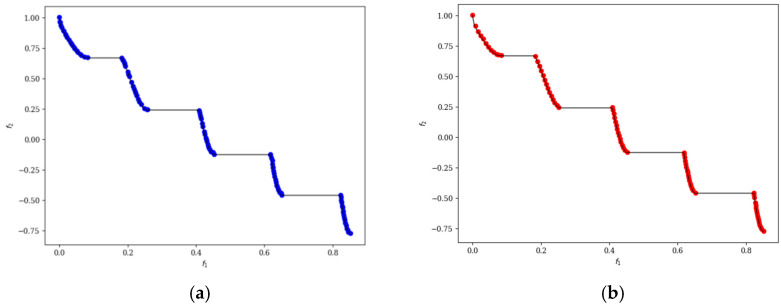
ZDT3 Pareto curve: (**a**) NSGA-II; (**b**) MOEDO.

**Figure 8:**
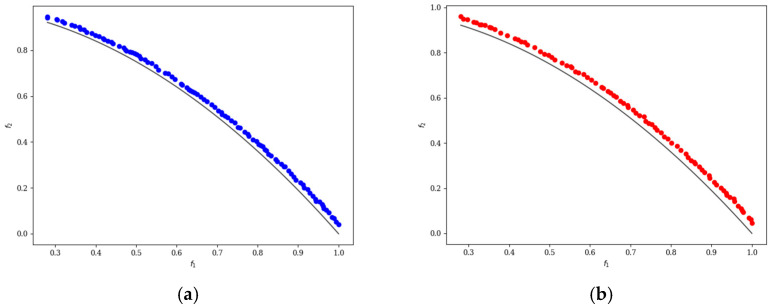
ZDT6 Pareto curve: (**a**) NSGA-II; (**b**) MOEDO.

**Figure 9:**
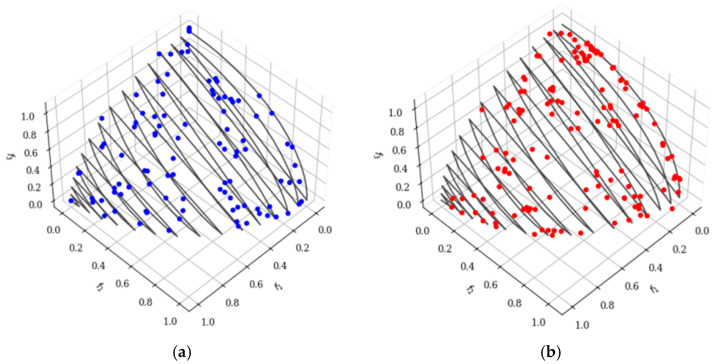
DTLZ2 Pareto curve: (**a**) NSGA-II; (**b**) MOEDO.

In the ten diagrams illustrated in Figure 5, Figure 6, Figure 7, Figure 8 and Figure 9, the black line represents the ideal Pareto front curve. The blue dot diagram on the left illustrates the Pareto front curves obtained by the classical NSGA-II algorithm across various test functions. It can generally be observed that these curves are close to the ideal Pareto curve; however, in some intervals, there are instances of crowding, stacking, and interruptions, indicating that the uniformity of individual distribution is not sufficiently good. In contrast, the red dot diagram on the right presents the front curves achieved by the MOEDO algorithm for different test functions, showing that the MOEDO algorithm seldom experiences crowding, stacking, or interruptions for both two-objective and three-objective test functions. This indicates that the MOEDO algorithm is better at avoiding local optima and exhibits superior uniformity.

### Comparison of Algorithm Performance Metrics

In the following study, three performance indicators were employed to assess the performance of the optimization algorithms: Hypervolume (HV), Inverted Generational Distance (IGD), and Spread (SD). The Wilcoxon signed-rank test for the counts of test problems exhibiting superior, inferior, and comparable performance of the respective optimization algorithms in addressing multi-objective issues was conducted. The primary aim of evaluating these four performance indicators is to comprehensively and objectively measure and compare the effectiveness of different optimization algorithms when solving multi-objective optimization problems. Each criterion reflects the performance characteristics of the algorithm from a distinct perspective.

(1) Hypervolume (HV)

Hypervolume (HV) is an important metric for evaluating the performance of multi-objective optimization algorithms. It measures the volume occupied by the set of solutions identified by the algorithm in the objective space. The core concept of the HV metric is to assess the coverage of the solution set relative to the ideal Pareto front; that is, the larger the solution set, the broader the area of the Pareto front it covers, indicating better algorithm performance.

(8) $$HV(S)=\int_{R^{m}}I_{y\in S}(y)dy$$

In Equation (8), S represents the set of solutions that approximate the Pareto front; *y* denotes the objective variable; *l_y_*_∈*S*_ denotes the indicator function, which is 1 if *y* is in the set *S* and 0 otherwise; and *R_m_* indicates the objective space m-dimensional.

HV is typically approximated using sampling points, as direct integration is infeasible in high-dimensional spaces. The optimization objective of the algorithm is to maximize the HV value, thereby showcasing the superiority and diversity of the set of solutions obtained by the algorithm in multi-objective optimization problems. Table 3 illustrates the final solution distribution of the MOEDO algorithm compared with MOMPA, NSGA-II, and MOEA/D when using the generational distance (GD) metric. Here, ‘+’ indicates that MOEDO significantly outperforms the comparison algorithms, ‘−’ indicates inferior performance, and ‘=’ indicates no significant difference. The judgment for these symbols is based on the Wilcoxon rank-sum test at a significance level of 0.05, a relative error range threshold where differences are considered significant if the *p*-value < 0.05: ‘+’ if the *p*-value < 0.05 and MOEDO achieves a lower GD value, better performance is achieved; ‘−’ if the *p*-value < 0.05 and MOEDO achieves a higher GD value, worse performance is obtained; and ‘=’ if the *p*-value ≥ 0.05, there is no significant difference within the acceptable index deviation threshold [9].

(2) Inverse Generational Distance (IGD)

Generational Distance (IGD) is a metric used to assess the performance of multi-objective optimization algorithms, primarily measuring the average distance between the solution set identified by the algorithm and the true Pareto front [10]. The calculation formula for IGD is as follows:

(9) $$IGD(P,Q)=\frac{\sum_{v\in P}d(v,Q)}{\left|P\right|}$$

In Equation (9), *P* is a set of points uniformly distributed on the true Pareto front, and |*P*| denotes the number of individuals in the point set distributed on the true Pareto front. Q represents the optimal Pareto solution set obtained by the algorithm. The term *d*(*v*, *Q*) describes the minimum Euclidean distance from individual *v* in *P* to the population *Q*.

Table 4 presents the final solution distribution when comparing the MOEDO algorithm with MOMPA, NSGA-II, and MOEA/D using the Inverted Generational Distance (IGD) indicator. In the ZDT benchmark tests, the MOEDO algorithm performs excellently, particularly in ZDT1, ZDT2, ZDT3, and ZDT6, showcasing outstanding average and standard deviation results. The results in the table demonstrate that MOEDO exhibits superior performance across all benchmark tests [11].

(3) Degree of Expansion SD

The Spacing Metric (SD) is an evaluation metric used to assess the performance of multi-objective optimization algorithms, primarily aimed at measuring the distribution of solutions within a solution set [12,13]. The SD metric reflects the uniformity of the solution set, indicating whether the distances between various points in the solution set are approximately equal. An ideal solution set should be uniformly distributed in the objective function space, without any clustered or sparse areas. The formula for calculating SD is as follows:

(10) $$SD=\sqrt{\frac{1}{m-1}\sum_{i=1}^{m}{\left(\frac{d_{i}-\bar{d}}{\max\{d_{max},\bar{d}\}}\right)}^{2}}$$

In Equation (10), *m* represents the number of solutions in the solution set; *d_i_* denotes the distance from the *i*-th solution to its nearest neighbor; *d* is the average distance from all solutions to their nearest neighbors; and *d_max_* represents the maximum value among all *d_i_*.

The closer the SD value is to 0, the more uniform the distribution of the solutions in the solution set, indicating better performance of the algorithm.

Table 5 presents the final solution distribution of the MOEDO, MOMPA, NSGA-II, and MOEA/D algorithms when using the SD. Among all metrics, the MOEDO algorithm demonstrates outstanding performance in terms of standard deviation and mean measures across ZDT1, ZDT2, ZDT3, ZDT4, and DTLZ2 [14].

## 5. Experimental Results and Analysis

**Figure 10:**
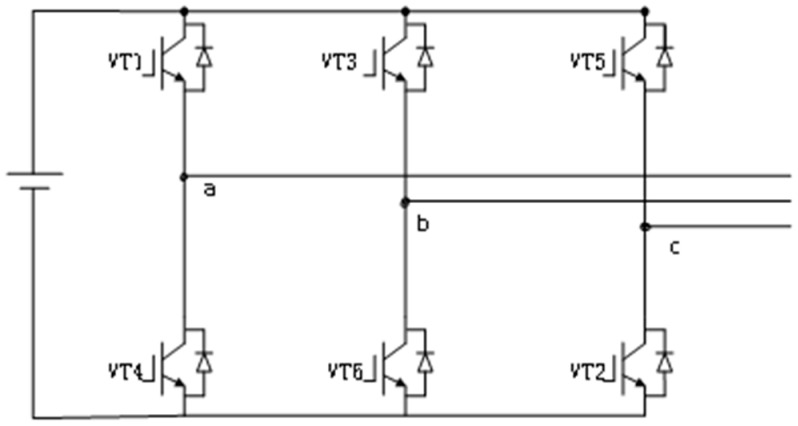
Three-phase full-bridge inverter circuit topology.

The three-phase full bridge inverter circuit consists of six IGBT devices, as shown in Figure 10. For a three-phase circuit, there are a total of three bridge arms in operation, which generate significant thermal losses during operation, primarily due to the conduction losses and switching losses of the IGBTs [15]. Therefore, to ensure the stability of the circuit and extend its lifespan, the three-phase full bridge inverter circuit often requires the installation of heat sinks and, when necessary, the use of fans to enhance cooling effectiveness.

For a two-level three-phase full-bridge inverter with a power rating of 10 kW, sintered wick straight heat pipes can be embedded into the upper portion of the conventional heat sink base to form a heat pipe heat sink. Heat pipes possess extremely high thermal conductivity, enabling rapid heat transfer from the evaporation section to the condensation section. Therefore, arranging the straight heat pipes directly beneath the heat source allows the heat generated by the IGBT chips to be swiftly dissipated across the entire base [16]. Moreover, thermal simulation results indicate that when the cross-section of the straight heat pipe is semicircular, the radiator achieves optimal heat dissipation performance and the IGBT chip attains the lowest maximum temperature; when the cross-section is square, the radiator exhibits the poorest heat dissipation performance and the IGBT chip reaches the highest maximum temperature. Based on the principle of optimal cooling performance, the semicircular cross-section is the preferred choice.

**Figure 11:**
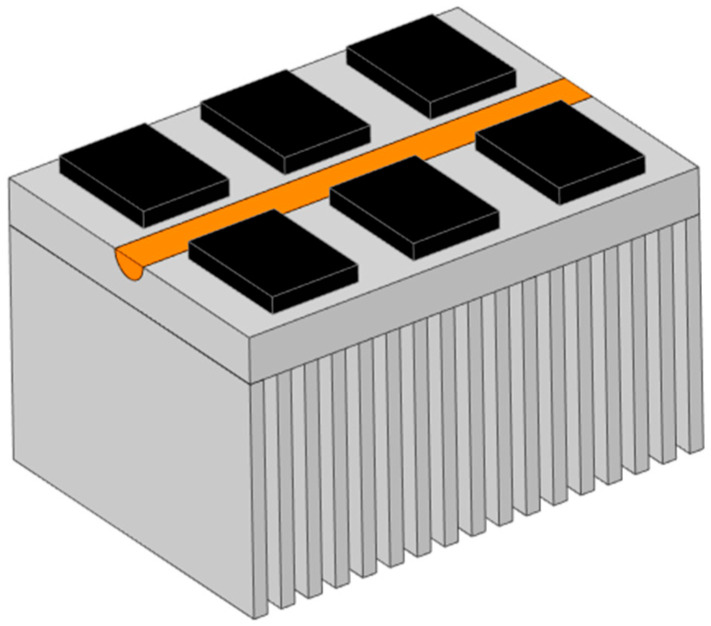
Heat pipe radiator 3D model.

The three-dimensional model of the heat pipe heat sink incorporating straight heat pipes is shown in Figure 11. The dimensions and shape of this three-dimensional model are identical to those of the conventional heat sink, with the sole distinction being the embedding of one sintered wick straight heat pipe within the base directly below the IGBT chips [17].

Due to the difficulty in directly simulating the complex phase-change heat-transfer process inside heat pipes using ANSYS Icepak, the heat pipes are modeled as an equivalent anisotropic thermal conductive medium. In typical ANSYS Icepak simulations for electronics cooling applications, the effective axial thermal conductivity is commonly set in the range of 20,000–30,000 W/K·m to represent the extremely high heat-transport capability driven by vapor–liquid phase change and capillary action in sintered wick heat pipes, while the radial thermal conductivity is set to be comparable to that of copper to account for conduction through the pipe wall and wick structure [18]. The value of 28,000 W/K·m was selected for the axial direction in this study as it falls within the validated range for high-performance straight/loop sintered wick heat pipes under natural/forced convection conditions, ensuring accurate prediction of temperature spreading across the baseplate and fins. The radial thermal conductivity was set to 400 W/K·m.

**Figure 12:**
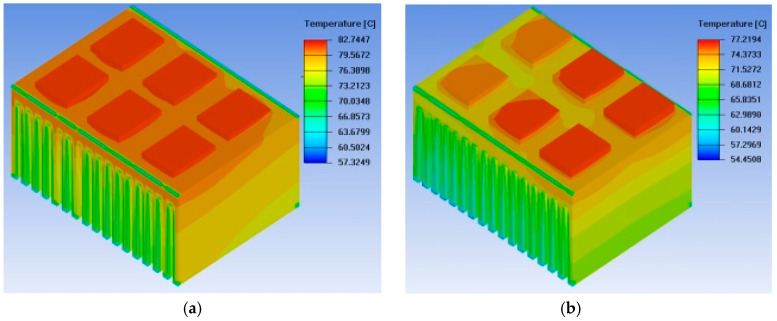
Temperature field distribution of traditional radiators and direct heat pipe radiators: (**a**) Traditional heat sink; (**b**) Straight heat pipe heat sin.

**Figure 13:**
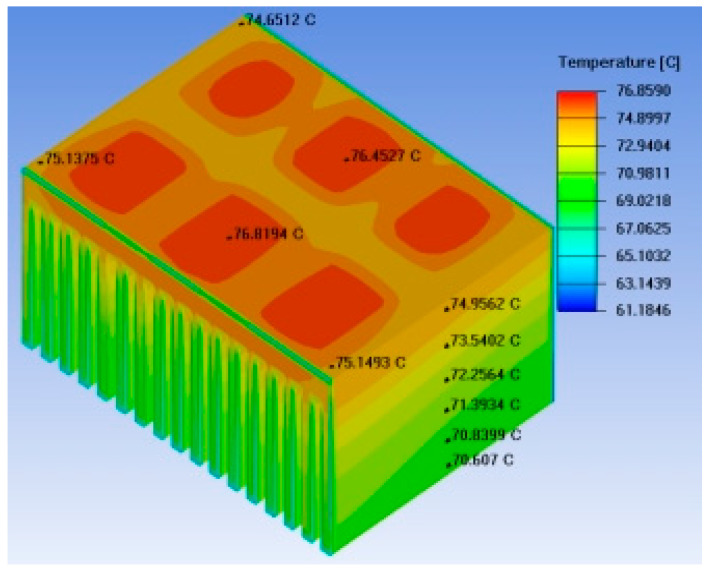
Thermal simulation results of the base and fins of the heat pipe radiator.

The temperature field distribution diagrams for the conventional heat sink and the straight heat pipe heat sink are shown in Figure 12, while the thermal simulation results for the base and fins of the straight heat pipe heat sink are presented in Figure 13. It can be observed that incorporating straight heat pipes into the conventional heat sink structure results in a certain degree of reduction in the heat source temperature, achieving a uniform temperature distribution across the heat sink base, with minimal variations in the overall base temperature [19]. However, the temperature distribution in the fin section is extremely uneven, with higher temperatures closer to the base; the temperature difference between the fin bottom and the fin top exceeds 5 °C, indicating that the heat generated by the IGBT chips is not sufficiently transferred to the lower portion of the fins, thus demonstrating that the heat dissipation capacity of the fins is not fully utilized.

**Figure 14:**
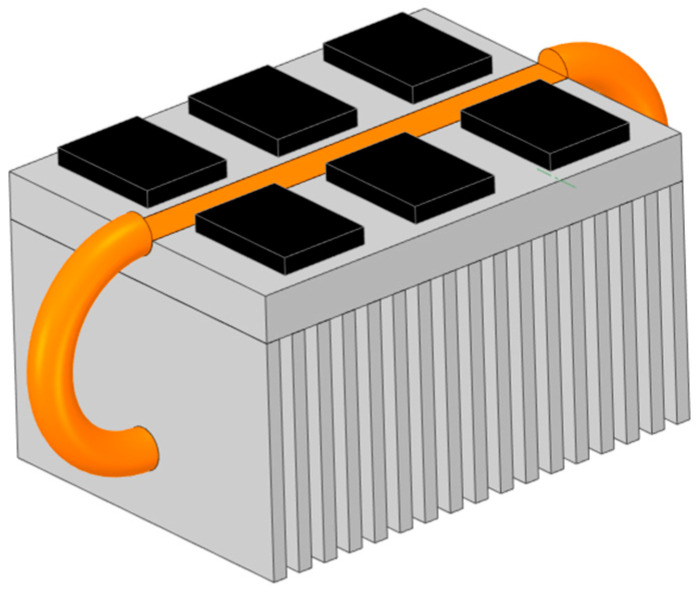
The three-dimensional model of the loop heat pipe heat sink.

To fully exploit the heat dissipation potential of the fins, this section adopts a profiled design approach for the heat pipes. A three-dimensional model of the loop heat pipe heat sink after the profiled heat pipe design is shown in Figure 14.

The loop heat pipe heat sink adopts a profiled heat pipe design, which enables the heat generated by the IGBT chips to be rapidly transferred through the heat pipes to the entire base plate and the lower half of the fins. According to the basic principles of heat transfer, this can be understood as the loop heat pipe heat sink reducing its total thermal resistance by adding a low-thermal-resistance thermal path in parallel with the original thermal path.

**Figure 15:**
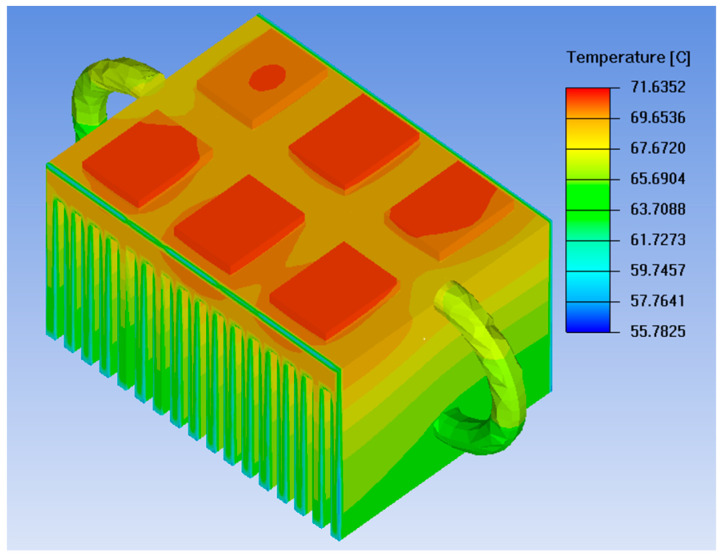
Loop heat pipe radiator thermal simulation.

After the three-dimensional model of the loop heat pipe heat sink is established, mesh generation is performed, followed by the application of identical settings for boundary conditions and material properties. The power loss of the IGBT chips, material types, and material properties are consistent with those in the conventional heat sink system [20]. External conditions such as fan air velocity and ambient temperature are also identical to the thermal simulation settings of the conventional heat dissipation system. Finally, the thermal simulation software ANSYS Icepak is employed to conduct a steady-state thermal simulation on the loop heat pipe heat sink. The thermal simulation results for the loop heat pipe heat sink are shown in Figure 15.

Observing the thermal simulation diagram reveals that the loop heat pipe heat sink achieves uniform temperature distribution in the heat sink fins, indicating that this design effectively transfers heat to the heat sink fins [21]. The maximum temperature of the IGBT chips is also reduced from 82.745 °C in the original straight heat pipe heat sink to 71.635 °C, with a temperature reduction of 11.11 °C. In summary, the profiled heat pipe design significantly enhances the heat dissipation capacity of the heat sink.

A thermal simulation and volume calculation were performed for the sample points selected in Table 1, with the results presented in Table 6.

From this, a multiple regression model can be derived, using the IGBT temperature and heatsink volume as response variables, as shown in Equations (10) and (11).

(11) $$\begin{array}{l} Y_{1}(x)=115.9019+0.4532x_{1}-0.364x_{3}+0.22x_{4}+1.0251x_{5}+1.5251x_{6}-0.0175x_{1}^{2}-0.128x_{2}^{2}-0.0663x_{3}^{2}\\ -0.0014x_{4}^{2}-0.0217x_{5}^{2}-0.0081x_{6}^{2}-0.0013x_{1}x_{4}-0.0385x_{1}x_{5}+0.0294x_{2}x_{4}-0.0106x_{2}x_{5}+0.0418x_{2}x_{6}\\ +0.0191x_{3}x_{4}+0.0142x_{3}x_{5}-0.0035x_{3}x_{6}+0.0141x_{4}x_{5}-0.0223x_{4}x_{6}+0.016x_{5}x_{6} \end{array}$$

(12) $$Y_{2}(x)=\left(\begin{array}{l} -5.7512+0.8105x_{1}+1.4397x_{2}-2.4975x_{3}+0.0277x_{4}+1.2591x_{5}+0.129x_{6}-0.0021x_{1}^{2}\\ -0.1842x_{2}^{2}+0.2103x_{3}^{2}+0.0003x_{4}^{2}-0.1800x_{5}^{2}-0.0001x_{6}^{2}-0.0181x_{1}x_{2}+0.0002x_{1}x_{3}\\ -0.00057x_{1}x_{4}-0.0099x_{1}x_{5}+0.0015x_{1}x_{6}+0.0537x_{2}x_{3}+0.0178x_{2}x_{4}-0.01977x_{2}x_{5}\\ -0.0038x_{2}x_{6}+0.0006x_{3}x_{4}-0.0383x_{3}x_{5}+0.0009x_{3}x_{6}+0.0150x_{4}x_{5}-0.0007x_{4}x_{6}\\ +0.0119x_{5}x_{6} \end{array}\right)\times 10^{5}$$

**Figure 16:**
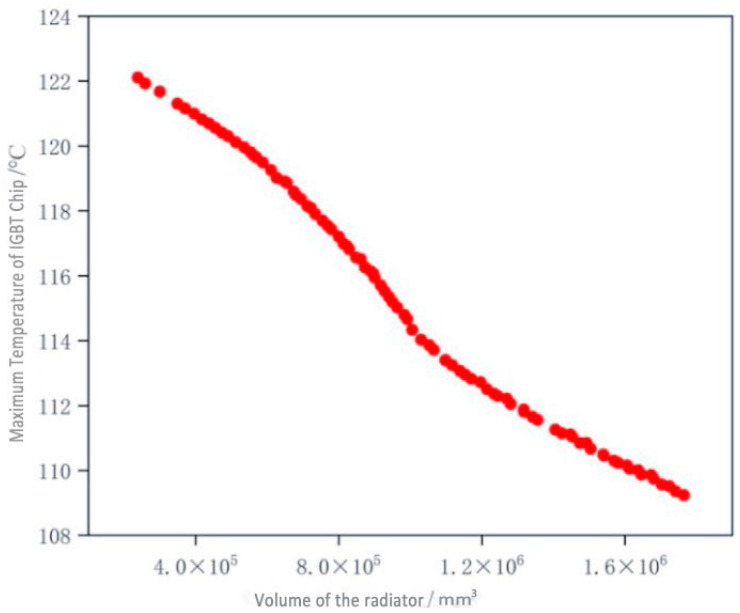
Pareto solution set of IGBT temperature and heat sink volume.

**Figure 17:**
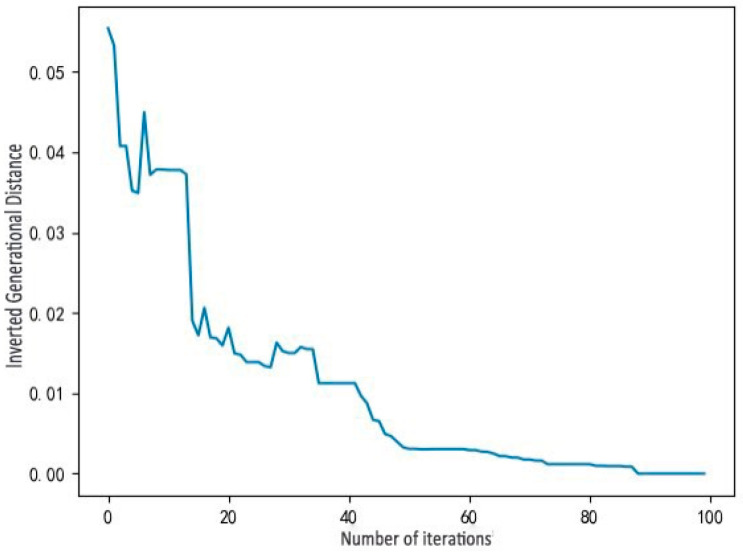
Inverted generational distance.

Based on the response surface models given in Equations (11) and (12), the maximum number of iterations is set to 100, and the population size is set to 100. The Pareto front obtained by the proposed MOEDO algorithm for minimizing the maximum IGBT temperature and heat sink volume of the three-phase power converter is shown in Figure 16. The convergence curve of the inverted generational distance (IGD) metric is depicted in Figure 17.

Based on the parameters specified in the selected IGBT module’s datasheet, the maximum allowable operating junction temperature is limited to 125 °C to extend device lifetime and reduce failure risk. Considering the variability of actual operating conditions and engineering requirements, the permissible temperature rise is constrained to no more than 12 °C [22]. Therefore, the system aims to keep the IGBT’s peak temperature below 113 °C. Following this screening logic, the optimal design solution was ultimately selected from the Pareto front. The corresponding design variables and objective values of the optimized heat dissipation system are presented in Table 7.

**Figure 18:**
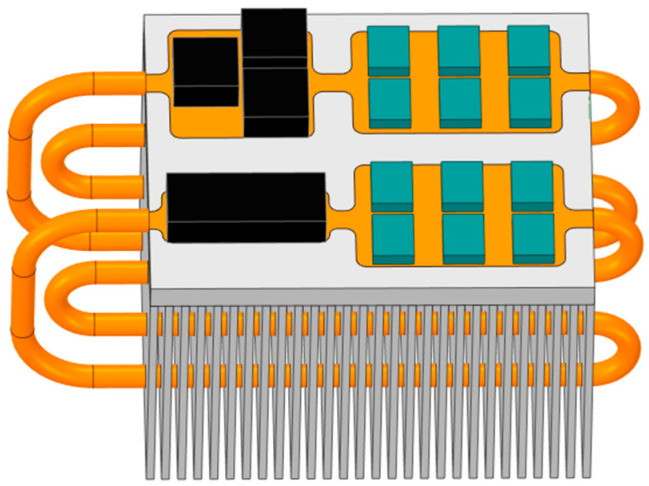
The overall structure after parameter optimization.

The overall structure of the cooling system for the bidirectional power converter, after parameter optimization, is shown in Figure 18.

**Figure 19:**
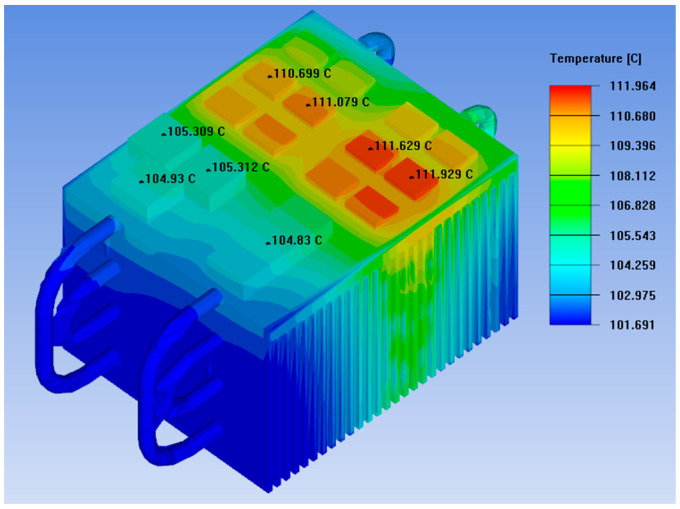
Physical design diagram of the radiator.

Based on the design variables listed in Table 7, the geometry was modeled and meshed, followed by thermal simulation in ANSYS Icepak. The physical design diagram of the radiator is shown in Figure 19.

The comparison of the results shows that under an ambient temperature of 100 °C, the optimized heat dissipation system reaches a maximum operating temperature of 111.964 °C after the bidirectional power converter has been running continuously for 8 h. This corresponds to a temperature rise of 11.964 °C, with a heat sink volume of 1,319,272.28 mm^3^. The performance of the traditional heat dissipation system is shown in Figure 19. Compared with the conventional design, the maximum temperature is reduced by 23.14 °C, representing a 17.12% decrease, and the heat sink volume is decreased by 225,727.72 mm^3^, corresponding to a 14.61% reduction. These results indicate that the parameter-optimized heat dissipation system not only enhances cooling capability but also achieves a noticeable reduction in heat sink mass.

## 6. Conclusions

This study addresses the multi-objective optimization problem of heat pipe heat sinks for bidirectional power converters, where cooling efficiency, volume, and weight must be simultaneously balanced under stringent engineering constraints. To overcome the limitations of traditional algorithms such as NSGA-II in convergence speed and local-optima entrapment, a Multi-Objective Exponential Distribution Optimizer MOEDO is developed by extending the single-objective Exponential Distribution Optimizer EDO through the integration of elite non-dominated sorting, crowding distance, and an information feedback mechanism. Combined with Optimal Latin Hypercube Sampling OLHS and a high-fidelity second-order response surface surrogate model, the proposed framework enables the efficient and accurate prediction of radiator volume and maximum IGBT junction temperature while significantly reducing computational cost.

Benchmark tests on ZDT1–ZDT6 and DTLZ2 functions demonstrate that MOEDO outperforms MOMPA, NSGA-II, and MOEA/D in Hypervolume HV, Inverted Generational Distance (IGD), and SD. Wilcoxon signed-rank test results confirm the statistically significant superiority of the majority of the evaluated cases. ANSYS Icepak simulations further validate the practical effectiveness: compared with the conventional design, the optimized heat pipe heat sink reduces the maximum IGBT temperature by 17.12 percent and decreases the heat sink volume by 14.61 percent. These improvements simultaneously enhance thermal performance and achieve lightweight, compact design without compromising structural integrity or manufacturability.

The proposed MOEDO-based optimization methodology provides both theoretical support and a practical pathway for the thermal management of high-power-density power electronics. With the rapid advancement of electrification in transportation and energy systems, bidirectional power converters are increasingly deployed in space-constrained applications such as electric vehicles, aircraft, and compact renewable energy systems. Effective thermal management in these scenarios is critical not only for reliability and efficiency but also for realizing miniaturization and lightweight design. The present work offers a robust and scalable solution that can be readily extended to other high-heat-flux power electronic devices.

## Figures and Tables

**Table 1:** Sample points of bidirectional power converter heat sink design variables.

Sample Point	Base Length (mm)	Base Width (mm)	Fin Thickness (mm)	Fin Spacing (mm)	HP Circumference (mm)	HP Radius (mm)
Sample Point 1	191.43	134.29	2.71	3.14	1908.29	3.64
Sample Point 2	168.57	142.86	2.86	2.14	1874	3.32
Sample Point 3	164.29	136.43	4	2	1942.57	4.34
Sample point 4	182.86	137.5	1.57	5	1891.14	4.07
Sample point 5	175.71	158.93	3.57	2.71	2019.71	3.43
Sample point 6	170	157.86	4.14	3.43	2054	4.39
Sample point 7	200	132.14	3.29	4	2011.14	3.48
Sample point 8	184.29	148.21	1.86	4.29	1839.71	3.38
Sample point 9	180	154.64	3	1.29	1994	3.54
Sample point 10	171.43	135.36	1.29	3.71	1822.57	4.23
Sample point 11	167.14	140.71	3.86	3.29	1959.71	3.91
Sample point 12	190	145	4.57	1.71	2002.57	3.16
Sample point 13	195.71	143.93	4.43	4.86	1951.14	4.02
Sample point 14	198.57	150.36	2	3	1976.86	4.18
Sample point 15	197.14	149.29	2.57	3.86	1882.57	3.27
Sample point 16	187.14	160	5	2.29	1856.86	4.29
Sample point 17	181.43	151.43	4.86	1.86	2045.43	3.59
Sample point 18	161.43	156.79	1.43	2.86	2036.86	3.86
Sample point 19	192.86	131.07	4.29	4.14	1916.86	3.8
Sample point 20	162.86	155.71	2.14	4.43	1968.29	3.21
Sample point 21	165.71	139.64	1.71	4.57	1925.43	3.11
Sample point 22	185.71	133.21	3.43	4.71	1848.29	4.13
Sample point 23	194.29	152.5	4.71	1.57	1934	3.05
Sample point 24	178.57	153.57	3.14	1.43	2028.29	3.96
Sample point 25	172.86	146.07	2.29	2.43	1985.43	3.7
Sample point 26	188.57	147.14	3.71	3.57	1831.14	4.45
Sample point 27	174.29	138.57	2.43	1.14	1899.71	3.75
Sample point 28	177.14	141.79	1.14	2.57	1865.43	4.5

**Table 2:** Test functions.

Test Function	Function Formula	Constraints
ZDT1	$f_{1}(x_{1})=x_{1}$ $f_{2}(x_{1})=g\times (1-\sqrt{f_{1}/g})$ $g(x)=1+9\sum_{i=2}^{m}x_{i}/(m-1)$	$m=30;0\leq x_{i}\leq 1$
ZDT2	$f_{1}(x_{1})=x_{1}$ $f_{2}(x_{1})=g\times (1-{\left(f_{1}/g\right)}^{2})$ $g(x)=1+9\sum_{i=2}^{m}x_{i}/(m-1)$	$m=30;0\leq x_{i}\leq 1$
ZDT3	$f_{1}(x_{1})=x_{1}$ $f_{2}(x_{1})=g\times \left(1-\sqrt{f_{1}/g}-(f_{1}/g)\sin(10\pi f_{1})\right)$ $g(x)=1+9\sum_{i=2}^{m}x_{i}/(m-1)$	$m=30;0\leq x_{i}\leq 1$ $-5\leq x_{i}\leq 5,i=2,3,\ldots ,m$
ZDT6	$f_{1}(x_{1})=1-\exp(-4x_{1})\sin^{6}(6\pi x_{1})$ $f_{2}(x_{1})=g\times (1-{\left(f_{1}/g\right)}^{2})$ $g(x)=1+9{\left(\sum_{i=2}^{m}x_{i}/(m-1)\right)}^{0.25}$	$m=30;0\leq x_{i}\leq 1$
DTLZ2	$f_{1}(x_{1})=(1+g)\cos(x_{1}\pi /2)\cos(x_{2}\pi /2)$ $f_{2}(x_{1})=(1+g)\cos(x_{1}\pi /2)\sin(x_{2}\pi /2)$ $f_{3}(x_{1})=(1+g)\sin(x_{1}\pi /2)$ $g(x)=\sum_{i=3}^{m}{\left(x_{i}-0.5\right)}^{2}$	$m=12;0\leq x_{i}\leq 1$

**Table 3:** HV measurement results of different algorithms on ZDT and DTLZ test functions.

Problem	M	MOEDO	MOMPA	NSGA-II	MOEA/D
ZDT1	2	8.6894 × 10^−1^ (3.27 × 10^−3^) +	7.0827 × 10^−1^ (5.62 × 10^−2^) −	8.3442 × 10^−1^ (3.08 × 10^−2^) =	8.3849 × 10^−1^ (1.68 × 10^−2^) =
ZDT2	2	5.0055 × 10^−1^ (6.08 × 10^−2^) +	3.0232 × 10^−1^ (8.18 × 10^−2^) −	3.1603 × 10^−1^ (6.09 × 10^−2^) −	3.6941 × 10^−1^ (1.53 × 10^−1^) =
ZDT3	2	9.2335 × 10^−1^ (1.28 × 10^−1^) =	7.8164 × 10^−1^ (8.15 × 10^−2^) −	9.8307 × 10^−1^ (3.86 × 10^−2^) =	9.4019 × 10^−1^ (4.36 × 10^−2^) =
ZDT6	2	4.3088 × 10^−1^ (3.53 × 10^−4^) =	3.2899 × 10^−1^ (3.00 × 10^−2^) −	4.2327 × 10^−1^ (6.42 × 10^−3^) −	4.1510 × 10^−1^ (5.64 × 10^−3^) −
DTLZ2	2	4.2015 × 10^−1^ (1.04 × 10^−4^) +	4.1779 × 10^−1^ (1.04 × 10^−3^) +	4.2006 × 10^−1^ (2.29 × 10^−5^) +	4.1885 × 10^−1^ (1.61 × 10^−4^) +
	3	7.3819 × 10^−1^ (1.81 × 10^−3^) +	7.4349 × 10^−1^ (5.65 × 10^−4^) +	7.4399 × 10^−1^ (1.66 × 10^−4^) +	7.4192 × 10^−1^ (1.19 × 10^−3^) +

**Table 4:** IGD measurement results of different algorithms on ZDT and DTLZ test functions.

Problem	M	MOEDO	MOMPA	NSGA-II	MOEA/D
ZDT1	2	3.5899 × 10^−5^ (1.66 × 10^−5^) +	9.6778 × 10^−3^ (2.99 × 10^−3^) −	8.2443 × 10^−4^ (3.28 × 10^−4^) −	1.0291 × 10^−3^ (3.16 × 10^−4^) −
ZDT2	2	5.5879 × 10^−5^ (1.90 × 10^−5^) +	6.8841 × 10^−3^ (3.43 × 10^−3^) −	5.5661 × 10^−4^ (2.37 × 10^−4^) −	9.6730 × 10^−4^ (7.50 × 10^−4^) =
ZDT3	2	3.1051 × 10^−5^ (8.12 × 10^−6^) +	1.7320 × 10^−2^ (9.64 × 10^−3^) −	4.1266 × 10^−4^ (1.23 × 10^−4^) −	4.3901 × 10^−3^ (3.51 × 10^−3^) −
ZDT6	2	4.3546 × 10^−5^ (2.85 × 10^−5^) +	1.3273 × 10^−2^ (4.57 × 10^−3^) −	7.6474 × 10^−4^ (4.63 × 10^−4^) −	1.5860 × 10^−3^ (5.35 × 10^−4^) −
DTLZ2	2	6.5895 × 10^−6^ (6.96 × 10^−7^) +	2.9149 × 10^−4^ (1.13 × 10^−4^) +	8.2707 × 10^−6^ (2.05 × 10^−6^) +	1.1447 × 10^−4^ (2.90 × 10^−5^) +
	3	5.9406 × 10^−4^ (2.19 × 10^−5^) +	5.4216 × 10^−4^ (3.69 × 10^−5^) +	5.1050 × 10^−4^ (4.98 × 10^−6^) +	1.1220 × 10^−3^ (7.88 × 10^−5^) +

**Table 5:** SD measurement results of different algorithms on ZDT and DTLZ test functions.

Problem	M	MOEDO	MOMPA	NSGA-II	MOEA/D
ZDT1	2	4.4163 × 10^−1^ (6.17 × 10^−2^) +	6.8453 × 10^−1^ (8.99 × 10^−2^) −	5.9068 × 10^−1^ (1.00 × 10^−1^) =	5.8964 × 10^−1^ (1.41 × 10^−1^) =
ZDT2	2	5.1648 × 10^−1^ (1.41 × 10^−1^) +	8.2618 × 10^−1^ (1.18 × 10^−1^) =	8.8220 × 10^−1^ (6.38 × 10^−2^) −	8.4278 × 10^−1^ (2.14 × 10^−1^) =
ZDT3	2	6.1982 × 10^−1^ (1.53 × 10^−1^) =	7.0831 × 10^−1^ (6.73 × 10^−2^) =	6.8544 × 10^−1^ (9.53 × 10^−2^) =	8.9016 × 10^−1^ (1.20 × 10^−1^) −
ZDT6	2	3.6182 × 10^−1^ (4.12 × 10^−2^) −	5.5838 × 10^−1^ (7.53 × 10^−2^) −	3.1380 × 10^−1^ (1.19 × 10^−1^) −	2.9883 × 10^−1^ (6.18 × 10^−2^) −
DTLZ2	2	1.4097 × 10^−1^ (2.12 × 10^−2^) +	1.9901 × 10^−1^ (2.84 × 10^−2^) +	1.9714 × 10^−1^ (3.81 × 10^−3^) +	7.0152 × 10^−1^ (2.50 × 10^−2^) =
	3	8.5844 × 10^−2^ (8.67 × 10^−3^) +	1.7089 × 10^−1^ (6.60 × 10^−4^) +	1.7596 × 10^−1^ (5.27 × 10^−3^) +	4.5225 × 10^−1^ (3.65 × 10^−2^) +

**Table 6:** IGBT temperature and heat sink volume at each sample point.

Sample Point	IGBT Temp (°C)	Heat Sink Vol (mm^3^)
Sample Point 1	115.4	1,387,846.083
Sample Point 2	113.7	1,558,910.455
Sample Point 3	112.6	1,682,075.098
Sample Point 4	124.6	807,049.24
Sample Point 5	111.2	1,797,973.539
Sample Point 6	112.9	1,701,794.031
Sample Point 7	117.7	1,407,039.408
Sample Point 8	122.3	1,055,391.458
Sample Point 9	107.6	2,155,940.571
Sample Point 10	122.1	771,361.2456
Sample Point 11	116.5	1,467,770.466
Sample Point 12	106.1	2,258,286.737
Sample Point 13	114.0	1,610,835.963
Sample Point 14	117.3	1,428,698.416
Sample Point 15	117.8	1,411,884.898
Sample Point 16	104.8	2,306,774.082
Sample Point 17	107.1	2,189,080.485
Sample Point 18	122.7	1,035,466.112
Sample Point 19	116.4	1,472,106.574
Sample Point 20	122.9	1,018,862.693
Sample Point 21	124.6	808,661.866
Sample Point 22	120.7	1,226,007.214
Sample Point 23	109.2	2,452,497.557
Sample Point 24	108.4	2,085,583.949
Sample Point 25	117.6	1,417,757.729
Sample Point 26	113.8	1,625,998.338
Sample Point 27	111.0	1,820,228.213
Sample Point 28	123.8	958,598.8865

**Table 7:** Design and objective variables of the optimized heat dissipation system.

Design and Objective Variables	Optimized Heat Dissipation System
Base Length (mm)	164.79
Base Width (mm)	142.25
Fin Thickness (mm)	3
Fin Spacing (mm)	3.2
Heat Pipe Perimeter (mm)	1962.74
Heat Pipe Radius (mm)	4.5
Heat Pipe Radius (mm)	2
Number of Heat Pipes	111.823
Heat Sink Volume (mm^3^)	1,319,272.28

## Data Availability

The data that support the findings of this study are available from the corresponding author upon reasonable request.

## Nomenclature

*a*_0_	Intercept term
*a*_1_	Baseplate length (mm)
*a*_2_	Baseplate width (mm)
*b*	Fin thickness (mm)
*b* *ᵢ*	Linear coefficient in response surface model
*c*	Total heat pipe perimeter (mm)
*c* *_ᵢⱼ_*	Interaction coefficient in response surface model
*CD* *ᵢ*	Crowding distance of the *i*-th individual
*d*	Fin spacing (mm)
*d* *ᵢ*	Quadratic coefficient
*f* *ₘ*	Multi Objective Marine Predators Algorithm
*i*	Non Dominated Sorting
*j*	Optimal Latin Hypercube Sampling
*k*	Spacing Distance
*m*	The number of solutions in the solution set
*M*	Number of objectives
*N*	Population size
*N*_min_	Number of design variables
*r*	Heat pipe radius (mm)
*S*	Set of solutions approximating the Pareto front
T_max_	Maximum IGBT temperature (°C)
$U_{i}^{t},$ $\;U_{i}^{t+1}$	Candidate individual from the EDO algorithm and the parent population
*xᵢ*, *xⱼ*	*i*-th and *j*-th components of the input variable
$x_{k}^{t}$	*k*-th individual selected from generation t
Greek letters	
α	Adaptive parameter/random threshold in EDO
∂_1_, ∂_2_	Dynamic weighting coefficient
σ	Standard deviation
σ^2^	Variance term in EDO
φ	Random number uniformly distributed in [0, 1]
Abbreviations	
CD	Crowding Distance
DTLZ	Deb–Thiele–Laumanns–Zitzler test
EDO	Exponential Distribution Optimizer
HV	Hypervolume
IFM	Information Feedback Mechanism
IGBT	Insulated Gate Bipolar Transistor
IGD	Inverse Generational Distance
MOEA/D	Multi-Objective Evolutionary Algorithm based on Decomposition
MOEDO	Multi-Objective Exponential Distribution Optimizer
MOMPA	Multi Objective Marine Predators Algorithm
NDS	Non Dominated Sorting
OLHS	Optimal Latin Hypercube Sampling
RMSE	Root Mean Square Error
RSM	Response Surface Methodology
SD	Spacing Distance
ZDT	Zitzler–Deb–Thiele test

## References

[1] Chen X., Wang Y., Liu Z., Zhang H., Li Q. (2024). Thermal management of high-power bidirectional DC–DC converter for electric vehicles using liquid cooling. Appl. Therm. Eng..

[2] Elghool A., Basrawi F., Ibrahim T.K., Habib K., Ibrahim H., Idris D.M.N. (2020). A multi-objective optimization to enhance the performance of thermo-electric generator combined with heat pipe-heat sink. Energy.

[3] Zhang Q., Li H. (2007). MOEA/D: A multiobjective evolutionary algorithm based on decomposition. IEEE Trans. Evol. Comput..

[4] Zhang Z., Li Y., Wang J., Liu H., Zhang Q. (2021). Multi-objective optimization of composite heat dissipation channel configuration for a three-dimensional trapezoidal heat-generating body. Appl. Therm. Eng..

[5] Mann G.W., Eckels S.J. (2019). Multi-objective optimization of heat transfer and pressure drop in helically coiled tubes using genetic algorithm. Int. J. Heat Mass Transfer.

[6] Zhang C., Liu W., Wang H., Li X., Zhang Y. (2022). Thermal resistance network analysis and simulation verification of heat sink design variables for bidirectional power converter. J. Eng. Appl. Sci..

[7] Crombecq K., Laermans E., Dhaene T. (2011). Efficient space-filling and non-collapsing sequential design strategies for simulation-based modeling. Eur. J. Oper. Res..

[8] Garud S.S., Karimi I.A., Kraft M. (2017). Design of computer experiments: A review. Comput. Chem. Eng..

[9] Wilcoxon F. (1945). Individual Comparisons by Ranking Methods. Biom. Bull..

[10] Mortazavi H., Montazeri S. (2024). Three-objective optimization of a novel solar-driven ejector enhanced refrigeration system. Energy Convers. Manag..

[11] Mi S., Li X., Wang Z., Zhang Y., Liu J. (2024). Multi-objective optimization of helical coil heat exchanger using ice slurry as the cooling medium via response surface methodology. Appl. Therm. Eng..

[12] Feng H., Chen Y., Zhang L., Chen L., Song Z. (2022). Configuration design and optimization of cooling channel with sidewall ribs and cavities for a three-dimensional rectangular heat-generating body. Int. Commun. Heat Mass Transfer.

[13] Sun K., Li J., Wang Z., Liu Y., Zhang H. (2022). Configuration design of semicircular sidewall rib cooling channel for a three-dimensional rectangular heat-generating body. Case Stud. Therm. Eng..

[14] Deb K., Pratap A., Agarwal S., Meyarivan T. (2002). A fast and elitist multiobjective genetic algorithm: NSGA-II. IEEE Trans. Evol. Comput..

[15] Ge Y., Liu Z., Sun H., Liu W. (2018). Optimal design of a segmented thermoelectric generator based on three-dimensional numerical simulation and multi-objective genetic algorithm. Energy.

[16] Mortazavi H., Beni H.M., Nadooshan A.A., Islam M.S., Ghalambaz M. (2024). 4E analysis and triple objective NSGA-II optimization of a novel solar-driven combined ejector-enhanced power and two-stage cooling (EORC-TCRC) system. Energy.

[17] Mi S., Liu J., Cai L., Xu C. (2024). Multi-objective optimization of two-phase ice slurry flow and heat transfer characteristics in helically coiled tubes with RSM and NSGA-II. Int. J. Therm. Sci..

[18] Mann G.W., Eckels S.J. (2019). Multi-objective heat transfer optimization of 2D helical micro-fins using NSGA-II. Int. J. Heat Mass Transfer.

[19] Zhang Z., Feng H., Chen L., Ge Y. (2021). Multi-objective constructal design for compound heat dissipation channels in a three-dimensional trapezoidal heat generation body. Int. Commun. Heat Mass Transfer.

[20] Sun K., Feng H., Chen L., Ge Y. (2022). Constructal design of a cooling channel with semi-circular sidewall ribs in a rectangular heat generation body. Int. Commun. Heat Mass Transfer.

[21] Feng H., Sun K., Chen L., Ge Y. (2023). Constructal design of a nanofluid cooling channel with sidewall ribs and cavities in a rectangular heat generation body. Case Stud. Therm. Eng..

[22] Zhang Q.Q., Yang J.H., Liu Z.C., Ma B.B., Guan X.B. (2023). Thermal performance study of high-power driver based on IGBT. Micro Mot..

